# The Clostridioides difficile Cysteine-Rich Exosporium Morphogenetic Protein, CdeC, Exhibits Self-Assembly Properties That Lead to Organized Inclusion Bodies in Escherichia coli

**DOI:** 10.1128/mSphere.01065-20

**Published:** 2020-11-18

**Authors:** A. Romero-Rodríguez, S. Troncoso-Cotal, E. Guerrero-Araya, D. Paredes-Sabja

**Affiliations:** a Microbiota-Host Interactions and Clostridia Research Group, Departamento de Ciencias Biológicas, Facultad de Ciencias de la Vida, Universidad Andrés Bello, Santiago, Chile; b ANID—Millennium Science Initiative Program, Millennium Nucleus in the Biology of the Intestinal Microbiota, Santiago, Chile; c Department of Biology, Texas A&M University, College Station, Texas, USA; University of Iowa

**Keywords:** *Clostridioides* (*Clostridium*) *difficile*, self-organization, spores, CdeC, exosporium

## Abstract

Clostridioides difficile is an obligately anaerobic, spore-forming, Gram-positive pathogenic bacterium that is considered the leading cause of nosocomial diarrhea worldwide. Recent studies have attempted to understand the biology of the outermost layer of C. difficile spores, the exosporium, which is believed to contribute to early interactions with the host. The fundamental role of the cysteine-rich proteins CdeC and CdeM has been described. However, the molecular details behind the mechanism of exosporium assembly are missing. The underlying mechanisms that govern exosporium assembly in C. difficile remain poorly studied, in part due to difficulties in obtaining pure soluble recombinant proteins of the C. difficile exosporium. In this work, we observed that CdeC was able to form organized inclusion bodies (IBs) in Escherichia coli filled with lamella-like structures separated by an interspace of 5 to 15 nm; however, CdeC expression in an E. coli strain with a more oxidative environment led to the loss of the lamella-like organization of CdeC IBs. Additionally, dithiothreitol (DTT) treatment of CdeC inclusion bodies released monomeric soluble forms of CdeC. Deletions in different portions of CdeC did not affect CdeC’s ability to aggregate and form oligomers stable under denaturation conditions but affected CdeC’s self-assembly properties. Overall, these observations have important implications in further studies elucidating the role of CdeC in the exosporium assembly of C. difficile spores.

**IMPORTANCE** The endospore of Clostridioides difficile is the vehicle for transmission and persistence of the pathogen, and, specifically, the exosporium is the first contact between the host and the spore. The underlying mechanisms that govern exosporium assembly in C. difficile remain understudied, in part due to difficulties in obtaining pure soluble recombinant proteins of the C. difficile exosporium. Understanding the exosporium assembly’s molecular bases may be essential to developing new therapies against C. difficile infection.

## Abstract (precis)

The endospore of Clostridioides difficile is the vehicle for transmission and persistence of the pathogen, and, specifically, the exosporium is the first contact between the host and the spore. The underlying mechanisms that govern exosporium assembly in C. difficile remain understudied, in part due to difficulties in obtaining pure soluble recombinant proteins of the C. difficile exosporium. Understanding the exosporium assembly’s molecular bases may be essential to developing new therapies against C. difficile infection.

## INTRODUCTION

Clostridioides difficile is an obligately anaerobic, spore-forming, Gram-positive pathogenic bacterium that is considered the leading cause of nosocomial diarrhea worldwide (1, 2). C. difficile infection (CDI) symptoms range from mild diarrhea to pseudomembranous colitis and toxic megacolon that can be life threatening. The CDI mortality rate is 5% and may increase to 20% in more severe cases (3). C. difficile spores can persist for long periods outside the host, facilitating transmission from host to host and resisting many environmental stressors (4). Therefore, spores are the vehicle for dispersion, transmission, and persistence. Recent reports have estimated the overall incidence rate of CDIs for patients of all ages was 2.24 per 1,000 admissions per year (5). The increased incidence and severity of CDI has led to a significant economic burden on health care systems due to the costs associated with treatment and extended stays of patients in the hospital (6, 7).

The spore surface is important for understanding how this pathogen interacts with the host and for the development of counter measurements. Recent studies have attempted to understand the biology of the outermost layer of C. difficile spores, the exosporium, which is believed to contribute to early interactions with the host (8–10). In a proteomic gel-free study, Díaz-González et al. demonstrated that the outermost exosporium layer of C. difficile spores has a total of 184 proteins, notably, 7 of which were previously characterized as spore coat and/or exosporium proteins, and 13 have been identified as uncharacterized proteins unique to C. difficile (11). The exosporium in the Bacillus cereus group has a basal layer containing cysteine-rich proteins, such as CotY and ExsY, which assemble into a porous hexagonal lattice and form “hair”-like protrusions from the basal layer (12–14). In the B. cereus group, the hair-like nap is formed mainly by radial projections of a highly collagen-like glycosylated protein, BclA, which is anchored in the basal layer (13). A group of structural proteins, which participate in the assembly of the exosporium of the B. cereus group, such as CotE, CotY, ExsA, ExsB, ExsY, and ExsM (15–18), has been described. In C. difficile, these proteins are absent in the exosporium proteome, but the BclA family’s orthologs have been identified. Besides, the cysteine-rich proteins CdeC and CdeM have been identified, with 9% and 8.7% of the amino acid content, respectively, which have a predominant role in the exosporium function and assembly (8, 19). For instance, the insertional inactivation of *cdeC* resulted in defective spore coat and exosporium assembly; the higher permeability to lysozyme increased the susceptibility to ethanol, heat, and macrophage inactivation (8). CdeM is necessary for the correct assembly of the exosporium (8). However, *cdeM* spores behaved as wild-type spores when measuring lysozyme’s permeability (8). The exosporium layer of C. difficile spores has a third cysteine-rich protein, CdeA, an 11.4-kDa uncharacterized protein encoded by CD2375 in strain 630 that contains 7.8% of cysteine-rich residues (11). CdeA exhibits a DUF1540 domain, but to our knowledge, no function has been assigned to this family; however, its distinctive signature is the presence of four conserved cysteines.

The underlying mechanisms that govern exosporium assembly in C. difficile remain understudied due to difficulties in obtaining pure soluble recombinant proteins of the C. difficile exosporium. We recently evaluated the effect of different heterologous Escherichia coli strains to increase soluble levels of proteins of the C. difficile spores (20). The results indicate that optimum soluble expression conditions may vary between 21, 30, and 37°C, depending on the protein, and at least CdeC, BclA1, and BclA3 required strains that provided an oxidative environment, such as E. coli SHuffle T7, for increased soluble levels. However, whether these observations can be applied to additional cysteine-rich exosporium proteins remains unclear.

In this work, while attempting to expand these findings to additional cysteine-rich proteins, CdeA and CdeM, we observed that only CdeC was able to form organized inclusion bodies in the E. coli BL21(DE3) pRIL strain, which was filled with lamella-like structures with an interspace of 5 to 15 nm. Moreover, we observed that these self-assembly properties were independent of the expression vector being used, the temperature, and the expression time. However, upon overexpression in the E. coli SHuffle T7 strain, the increased oxidative environment hampered CdeC’s self-organization ability. Moreover, dithiothreitol (DTT) treatment allows the release of soluble CdeC. Three truncated versions of CdeC were constructed to expand our analysis. The SDS-PAGE analysis revealed that all CdeC variants were able to aggregate, forming oligomers that were resistant to denaturation conditions, and transmission electron microscopy (TEM) analyses indicated that the self-organization properties of CdeC may expand across the protein. These observations have important implications in further studies related to the role of CdeC in exosporium assembly of C. difficile spores.

## RESULTS

### Cysteine-rich protein multimerizes during heterologous expression.

A recent study showed that the cysteine-rich protein CdeM exists not just as a monomer (predicted the molecular weight of 19 kDa) but also as several forms, including species of 25 and 60 kDa, presumably stabilized by the formation of disulfide bonds. CdeC was localized to the spore as a high-molecular-mass complex (21). Furthermore, upon heterologous expression in E. coli, CdeC is readily detectable as 42- and 84-kDa monomeric and dimeric immunoreactive species (21). Brito-Silva et al. (20) described optimized conditions for the overexpression of exosporium proteins CdeC, BclA1, BclA2, and BclA3 in an E. coli strain as a heterologous host. However, this work did not assess the formation of highly stable molecular complexes or aggregates.

**FIG 1:**
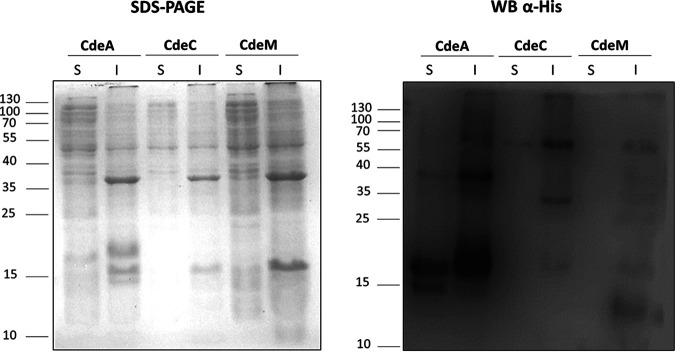
Heterologous overexpression of the cysteine-rich proteins CdeA, CdeC, and CdeM in soluble (S) and insoluble (I) fractions of E. coli lysates. Recombinant proteins were expressed in E. coli BL21(DE3) pRIL carrying plasmids pARR22, pARR10, and pARR21, respectively, and induced with 0.5 mM IPTG for 16 h at 37°C. The cells were disrupted in soluble or insoluble lysis buffer and electrophoresed in 15% SDS-PAGE gels; His-tagged immunoreactive proteins were detected by Western blotting as described in Materials and Methods. Each lane was loaded with 2 μg of protein lysate. Molecular mass (kDa) markers are indicated on the left.

Here, we expanded our previous work to look for the formation of complexes upon heterologous overexpression of the cysteine-rich proteins CdeA, CdeC, and CdeM, all of which were found in the exosporium proteome of C. difficile spores (11). To gain information on the multimerization in soluble and insoluble fractions, the *cdeA*, *cdeC*, and *cdeM* genes were cloned in the pETM11 vector and expressed in E. coli BL21(DE3) pRIL as described. Under the tested conditions (growth on LB broth supplements with glucose [LBG] at 37°C and induction with 0.5 mM isopropyl-β-D-thiogalactoside [IPTG]), SDS-PAGE analysis of soluble and insoluble fractions of CdeC and CdeM did not show clear abundant bands at the predicted molecular weights of the proteins CdeA (11.4 kDa), CdeC (44.7 kDa), and CdeM (19.2 kDa) (Fig. 1). For CdeA, two clear abundant bands of approximately 20 kDa and 38 kDa were detected in the soluble and insoluble fractions, probably corresponding to CdeA multimers. Also, in the insoluble fraction, a 60-kDa band was observed. In contrast, the soluble fraction exhibited an additional band of approximately 15 kDa, probably the protein’s monomer. For CdeC, Western blot analysis of the insoluble fraction revealed two clear His-immunoreactive bands (Fig. 1) of apparent molecular weights of 35 and 60 kDa.

On the other hand, Western blot analysis of the soluble fraction revealed an immunoreactive band at an apparent molecular weight of 60 kDa, presumably the monomeric form of CdeC. For CdeM, the Western blot analysis of the insoluble fraction showed four clearly immunoreactive bands of approximately 12, 19, 40, and 60 kDa. Those bands likely are the monomer and a multimerized form of CdeM (Fig. 1). The Western blot analysis of the soluble fraction revealed a faint immunoreactive band of an apparent molecular weight of 19 kDa, the putative monomeric form of CdeM. These results suggest that the three cysteine-rich proteins from the exosporium of C. difficile form multimers upon heterologous expression in E. coli, but this is particularly evident for CdeA and CdeM. The multimers are found mainly in the insoluble fraction, while the monomeric forms are found in the soluble fraction. For CdeC and CdeM, the soluble fraction bands are faint, suggesting the formation of inclusion bodies. Furthermore, the multimers formed by these proteins are highly stable and not dissembled by denaturing conditions present in the extraction buffer (20 mM Tris-HCl [pH 7.8], 500 mM NaCl, 5 mM imidazole, and 8 M urea) for the insoluble fraction. On the contrary, CdeA was abundant in both fractions.

### Cysteine-rich proteins CdeC and CdeM, but not CdeA, are found in large inclusion bodies.

**FIG 2:**
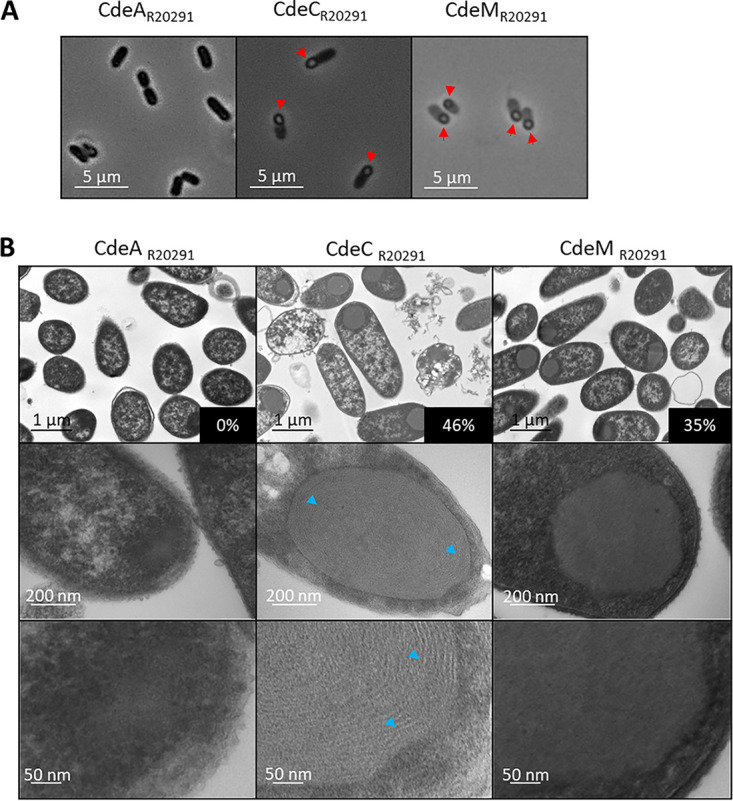
Microscopy analysis of E. coli cells expressing the cysteine-rich proteins CdeA, CdeC, and CdeM. Recombinant proteins were expressed in E. coli BL21(DE3) pRIL carrying plasmids pARR22, pARR10, and pARR21, respectively, and induced with 0.5 mM IPTG for 16 h at 37°C in LBG. (A) Samples were withdrawn from the cultures at 16 h and observed under phase-contrast microscopy. CdeC and CdeM examples of inclusion bodies are indicated with red arrowheads. (B) Electron microscopy analysis of E. coli BL21(DE3) pRIL cells expressing CdeA, CdeC, or CdeM (plasmids pARR22, pARR10, and pARR21, respectively). The samples were taken at 16 h postinduction, as previously mentioned. (Top) Representative micrographs of several E. coli cells. (Middle) Selected individual cells. (Bottom) Magnification of individual cells. Lamella-like formation in CdeC is indicated with blue arrowheads. The percentages in black boxes indicate the percentage of inclusion bodies found in each strain.

Due to the weak soluble expression of CdeC and CdeM proteins, cells overexpressing these proteins were observed under the microscope, and the formation of large inclusion bodies was detected for the cells expressing CdeC and CdeM but not CdeA (Fig. 2A). While the cells expressing CdeA looked like black bacilli (Fig. 2A), cells overexpressing CdeC and CdeM showed large inclusion bodies (IBs) that were observed as large refractive bodies predominantly located at one cell pole (Fig. 2A). To gain further information about the inclusion bodies’ putative structural features, cells expressing the recombinant proteins were observed by transmission electron microscopy (TEM). As expected, in the cytoplasm of the cells expressing CdeA, no clear inclusion bodies were detected (Fig. 2B); the magnification presented is to exemplify a cell with no IBs. Instead, in the case of CdeC- and CdeM-expressing cells, large rounded inclusion bodies were observed (Fig. 2B). For CdeC, 46% of the cells analyzed by TEM showed large inclusion bodies, while 35% of cells expressing CdeM formed IBs (Fig. 2B). These percentages are only an approximation, because the procedure for TEM analysis (particularly the microtome cuts) may bias or alter this number. Surprisingly, transmission electron micrographs revealed that in the case of CdeC, the ultrastructure of the inclusion bodies appeared as organized lamella-like structures (Fig. 2B). These results suggest that CdeC can form an organized ultrastructure, not just aggregates, as for CdeM.

### Inclusion bodies of CdeC, but not CdeM, exhibit a conserved organized structure.

The overexpression of *cdeC* from strain R20291 (*cdeC*_R20291_) in the vector pETM11 resulted in the generation of inclusion bodies. Nevertheless, unlike the inclusion bodies of CdeM, those of CdeC showed an organized lamella-like ultrastructure. Besides overexpression in pETM11, CdeC was also cloned and expressed in the vector pET22b, resulting in no differences in the lamella ultrastructure (see Fig. S1A in the supplemental material), indicating that localization of the His tag at the C terminus for pET22b and at both ends in the pETM11 construction did not affect the formation of lamella-like structures Also, the overexpression was tested at 21°C and 37°C; no differences in the lamella-like structure or the formation of oligomers were evidenced (Fig. S1B), indicating that both the self-organization of lamella-like structures and the formation of high-molecular-mass complexes are temperature independent.

10.1128/mSphere.01065-20.1 FIG S1 Effect of plasmid backbone and temperature of expression on the multimerization and ultrastructure of CdeC. (A) Effect of the plasmid on the CdeC ultrastructure. Recombinant proteins were expressed in E. coli BL21 (DE3) pRIL carrying the plasmids pARR19 and pARR10 expressing CdeC cloned in plasmid pET22b (left) or pETM11 (right), respectively. The cells were induced with 0.5 mM IPTG for 16 h at 37°C. (Top) Representative micrographs of several E. coli cells are shown. (Bottom) Selected individual cells. (B) Effect of the temperature on CdeC ultrastructure. Recombinant proteins were expressed in E. coli BL21 (DE3) pRIL carrying the plasmid pARR10. The cells were induced with 0.5 mM IPTG for 16 h at 21°C (left) or 37°C (right) in LBG. After incubation, samples were retrieved and prepared for TEM analysis, as described in Materials and Methods. (Top) Representative micrographs of several E. coli cells. (Bottom) Selected individual cells. (C) Effect of temperature on the soluble and insoluble expression of CdeC. Recombinant CdeC expression in E. coli BL21 (DE3) pRIL carrying pARR19 induced with 0.5 mM IPTG at 21°C (left) or at 37°C (right); samples at 30, 60, 90, 120, and 180 min postinduction were retrieved. Cells were collected and lysed in soluble (S) and insoluble (I) lysis buffers, electrophoresed, and analyzed by Western blotting as described in Materials and Methods. Each lane was loaded with 2 μg of protein lysate. Molecular mass (kDa) markers are indicated on the left. Download FIG S1, PDF file, 1.3 MB. Copyright © 2020 Romero-Rodríguez et al. 2020 Romero-Rodríguez et al. This content is distributed under the terms of the Creative Commons Attribution 4.0 International license.

The CdeC cysteine-rich proteins are highly conserved in *Peptostreptococcaceae* family members, and, at least in the epidemically relevant R20291 strain, it is essential for morphogenesis of the exosporium layer and spore resistance (8). We expanded the phylogenetic analysis previously reported (8); for this, the analysis of C. difficile genomes began with 1,835 multilocus sequence type (MLST)-assigned genomes (see Table S3). From these, 1,833 alleles of *cdeC* were extracted (see Table S5). In addition, 16 alleles of *cdeC*-like proteins with at least 90% coverage from the family *Peptostreptococcaceae* (*Clostridium* cluster XI) were added to extend the analysis to other species to discriminate amino acids at positions preserved throughout evolution. The assemblies with the access codes are described in Table S4. To reduce the data set without losing the diversity of the *cdeC*-like genes, they were clustered with 100% identity alleles. The information on the clusters obtained and the single alleles is described in Tables S3 and S4. The multiple alignments (see Fig. S2) of CdeC show interesting shared motifs among all the genes evaluated, which could be critical in the protein’s functionality. It should be noted that CdeC sequences belonging to C. difficile are very homogeneous among themselves, where the minimum identity at the amino acid level is greater than 90%, and so an analysis without outgroups would not account for indispensable structural domains of CdeC. Phylogenetic reconstructions using two different methodologies were topologically identical, suggesting a robust phylogenetic analysis (see Fig. S3).

10.1128/mSphere.01065-20.2 FIG S2 Sequence alignment of CdeC and its representatives from other species. The amino acids preserved in 100% of the analyzed sequences are highlighted. (A) Full-length alignment. (B) Subpart of panel A, including amino acids 142 to 242. (C) Includes amino acids in position 285 to 401. (D) Refers to amino acids in positions 449 to 495. Download FIG S2, PDF file, 6.7 MB. Copyright © 2020 Romero-Rodríguez et al. 2020 Romero-Rodríguez et al. This content is distributed under the terms of the Creative Commons Attribution 4.0 International license.

10.1128/mSphere.01065-20.3 FIG S3 Topological comparison of Bayesian (A) phylogenetic reconstructions using BEAST (B) and maximum likelihood reconstructions using RAxML. The branch coloration shows the Jaccard index, where 1 (blue) implies 100% similarity in the topology in a clade. All C. difficile strains fall into a monophyletic clade where their probability *a posteriori* is 100%. Interestingly, the closest common ancestor of CdeC is found in the ancestor between C. difficile and Clostridioides mangenotii, evidenced by the high sequence identity (61%), which forms in the Bayesian inference a monophyletic clade (probability *a posteriori* = 94.4%) between all C. difficile proteins and the two proteins studied (WP_024622166 and WP_027702509). Download FIG S3, PDF file, 0.1 MB. Copyright © 2020 Romero-Rodríguez et al. 2020 Romero-Rodríguez et al. This content is distributed under the terms of the Creative Commons Attribution 4.0 International license.

**FIG 3:**
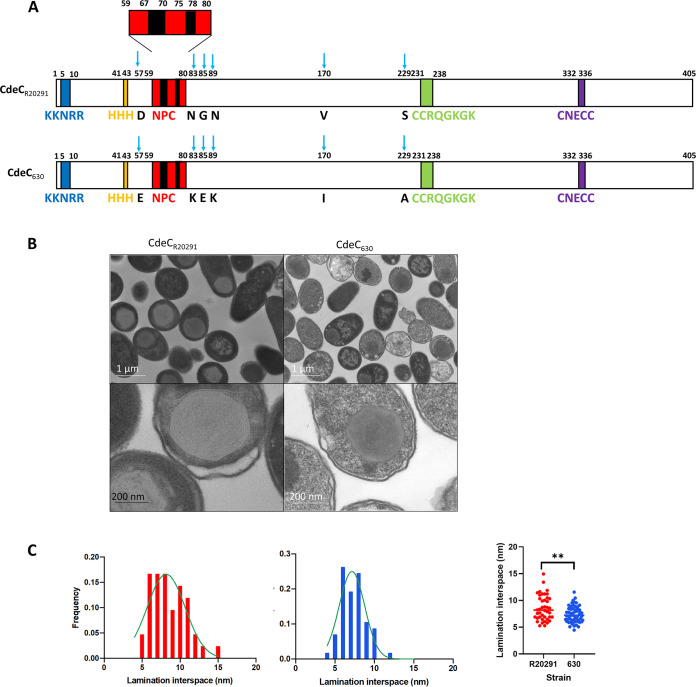
Amino acid conservation and ultrastructural organization of CdeC. (A) Schematic representation of the CdeC sequences of C. difficile strains 630 and R20291; each amino acid substitution is indicated by a blue arrow. (B) E. coli BL21(DE3) pRIL cells carrying plasmids expressing CdeC from C. difficile 630 (pDP339) or CdeC from R20291(pARR19) were induced with 0.5 mM IPTG for 16 h at 37°C. After induction, samples were retrieved from cultures and subjected to TEM analysis. (Top) Representative micrographs of several thin sections of E. coli BL21(DE3) pRIL expressing CdeC from C. difficile 630 or C. difficile R20291 are shown. (Bottom) Selected individual cells. (C) The distances between lamella-like structures were measured from at least 50 sections of inclusion bodies showing clear lamella-like structures, and the frequency distribution was plotted as a function of lamination interspace. The frequencies for lamination interspaces of CdeC strain 630 (blue) and CdeC strain R20291 (red) are shown. An unpaired *t* test analysis determined the statistical significance. **, *P* = 0.0023.

According to the secondary structure prediction of CdeC reported by Calderon-Romero et al. (8), CdeC contains the following sequence motifs (Fig. 3A): (i) near the N terminus of CdeC (NTD), there are two motifs of unknown function, namely, KKNKRR and three consecutive histidine residues, (ii) in the central region, there is a 6-NPC (Asn-Pro-Cys) repeat followed by two CCRQGKGK repeats; and (iii) in the C-terminal domain (CTD), a cysteine-rich sequence, CNECC, is found. This analysis was performed with the amino acid sequence of CdeC from strain 630. However, upon comparing the sequences of CdeC between strains R20291 and 630, we observed at least six amino acid substitutions (Fig. 3A). However, those changes are likely conservative substitutions, as in the case of aspartate 57 (negatively charged, polar, and hydrophilic) found in CdeC from strain 630 (CdeC_630_), while in the same position, a glutamate (negatively charged, polar, and hydrophilic) residue is present in CdeC_R20291_. Probably the most significant change, due to the chemical properties of the amino acids, is the presence of serine 229 (polar and noncharged) in CdeC_630_, while in the same position in CdeC_R20291_, the amino acid sequence includes an alanine (nonpolar and aliphatic).

Consequently, we asked whether the formation of the lamella is a feature that is conserved in CdeC. To address this, we compared the ultrastructures of CdeC_630_ and CdeC_R20291_ from strains 630 and R20291, which belong to clades sequence type 54 (ST54) MLST clade 1 and ST1 MLST clade 2, respectively. When observing cells from R20291 or 630 overexpressing CdeC by TEM, lamella-like formation was present in both strains (Fig. 3B). Consequently, the differences in those amino acid residues appear to be irrelevant for the self-organization of CdeC when expressed in the heterologous host, E. coli. Furthermore, we measure the distance between each lamination in individual inclusion bodies. For CdeC_630_, the distance between each lamella-like structure varied in the range of 5 to 12 nm, while for CdeC_R20291_, the distances were in the range of 5 to 15 nm. However, distances between lamellas with higher frequencies were 7 and 8 nm (Fig. 3C). These observations demonstrate that the properties of CdeC to form these self-assembled structures with well-defined laminations span across C. difficile clades. However, the molecular basis driving the self-organization that leads to a supramolecular structure is a matter for further studies.

### Effect of highly oxidizing cytoplasmic environment on the ultrastructure of CdeC inclusion bodies.

Several covalent cross-links, such as ε-(γ-glutamyl)-lysyl isopeptide bonds, stabilize the Bacillus subtilis spore architecture (22, 23). Considering the abundant cysteine residues in the CdeC amino acid sequence, it is feasible to hypothesize that at least part of the CdeC lamella-like self-assembly may be impacted by the formation of disulfide bonds, as is the case in some of the spore coat cysteine-rich proteins from B. subtilis (12). One approach to indirectly test the effect of the redox environment on the lamella formation is to use the E. coli SHuffle T7 strain. This strain has an oxidizing cytoplasm, contributing to the formation of stable disulfide bonds between cysteine residues of cytoplasmic proteins (24). Furthermore, this strain was also engineered to express DsbC in the cytoplasm, which isomerizes (rearranges) disulfide bonds to their native states. (24). During the oxidative folding of proteins, disulfide bonds are likely to form between those cysteine residues that are most proximal in the amino acid sequence. However, for the proper folding of the protein, those nascent disulfide bonds must rearrange (isomerize) to the half-cystine (the group formally derived by the removal of a hydrogen atom from the thiol group on the side chain of a cysteine) pairings of the native conformation (25).

**FIG 4:**
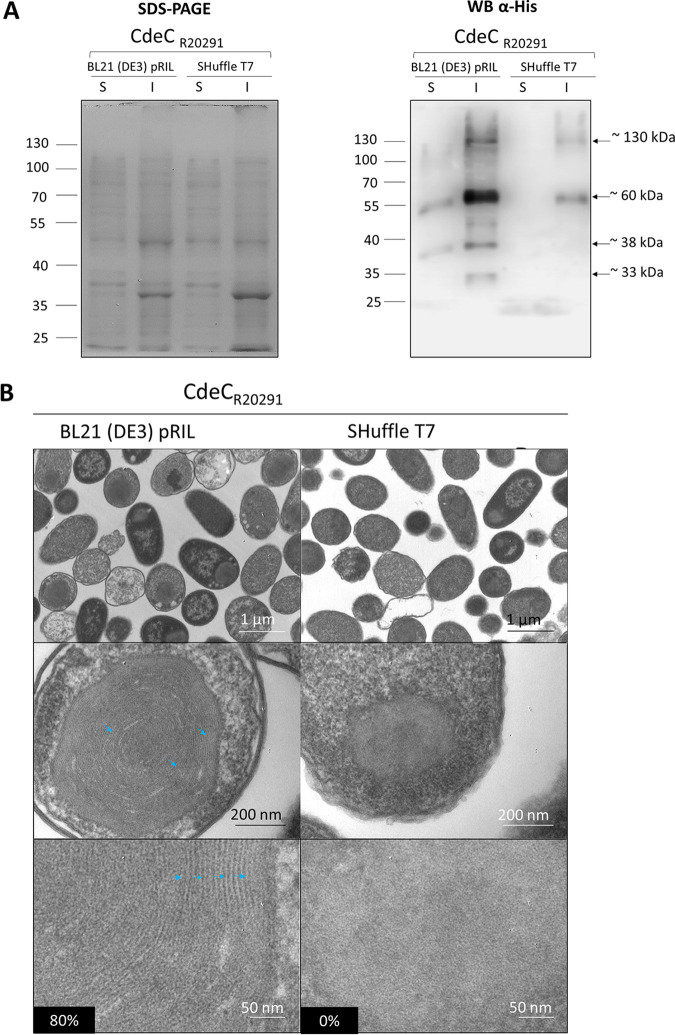
Effect of recombinant strain type in the soluble expression and structural organization of CdeC. (A) Recombinant CdeC expression in E. coli BL21(DE3) pRIL and SHuffle T7 strains carrying pARR19 induced with 0.5 mM IPTG for 16 h at 37°C. Cells were collected and lysed in soluble (S) and insoluble (I) lysis buffers, electrophoresed, and analyzed by Western blotting as described in Materials and Methods. Each lane was loaded with 2 μg of protein lysat**e**. Molecular mass (kDa) markers are indicated at the left, and molecular mass of the detected His-tagged immunoreactive bands are indicated at the right. (B) Thin sections of E. coli BL21(DE3) pRIL and E. coli SHuffle T7 expressing CdeC from C. difficile R20291 were analyzed by transmission electron microscopy as described in Materials and Methods. (Top) Representative micrographs of several E. coli cells are shown. (Middle) Selected individual cells. (Bottom) Magnified views of the thin sections of inclusion bodies inside E. coli BL21(DE3) pRIL or SHuffle T7. Lamella-like formation in CdeC is indicated with blue arrowheads. Numbers in the black boxes indicate the percentages of cells showing a lamella-like structure.

Consequently, to test the influence of an oxidizing cytoplasmic environment in forming multimeric species of CdeC, lysates prepared from E. coli BL21(DE3) pRIL and E. coli SHuffle T7 strains expressing CdeC were tested. Bands of approximately 35 and 60 kDa were observed in the soluble fraction of E. coli BL21(DE3) pRIL samples; the insoluble fraction showed the presence of three immunoreactive bands of approximately 35, 60, and 130 kDa (Fig. 4A). However, upon CdeC overexpression in E. coli SHuffle T7, no immunoreactive CdeC was detectable in soluble E. coli lysates, but two immunoreactive species of 60 and 130 kDa were evidenced in insoluble E. coli SHuffle T7 lysates. These results support the previous results (Fig. 1 and 2) that CdeC is poorly expressed in the soluble fraction regardless of the strain; the SHuffle strain does not improve the solubility of CdeC. Interestingly, the formation of a 130-kDa complex was observed in the insoluble fractions from both strains, suggesting that CdeC oligomerization is not affected by the cytoplasm’s oxidative state.

Next, we asked whether an oxidative cytoplasm affects the formation of inclusion bodies that are lamella like upon analyzing transmission electron micrographs. As aforementioned, 80% of inclusion bodies from E. coli BL21(DE3) pRIL displayed an organized lamella-like structure, while the E. coli SHuffle T7 inclusion bodies lost the lamella-like structure and were observed as amorphous aggregates (Fig. 4B). Overall, these results indicate that an excessively oxidizing environment, such as that encountered in E. coli SHuffle T7 strain, leads to the formation of CdeC aggregates probably as a result of unspecific cysteine bridges due to the high number of consecutive cysteines in the sequence of CdeC, suggesting that the self-assembly properties of CdeC depend on a subtle redox equilibrium.

### Effect of DTT on the ultrastructure of CdeC inclusion bodies.

Due to the formation of stable and high-density CdeC inclusion bodies, these can be isolated from cell lysates by differential centrifugation, providing fast, robust, and hence cost-efficient protocols to obtain large amounts of relatively pure protein. We first aimed to test whether CdeC inclusion bodies could be purified from E. coli lysates. For this, E. coli cells carrying CdeC inclusion bodies were lysed, and the remaining pellet was extracted with Triton X-100, a detergent that solubilizes membrane-associated material rather than aggregated proteins. After sequential washes with Triton X-100 and phosphate-buffered saline (PBS), the pellets resulted in an enrichment of CdeC inclusion bodies observed as dark spheres (see Fig. S4A). All the inclusion bodies presented immunoreactive fluorescence against anti-His antibodies, indicating the accessibility of the 6×His tag (Fig. S4A). Next, we asked if the purification protocol affects the ultrastructure of the bodies. For this, a sample of partially purified bodies was analyzed by TEM. Transmission electron micrographs showed that the purification protocol did not affect the lamella-like organization of the inclusion bodies (Fig. S4B). Therefore, these results indicate that CdeC inclusion bodies can be partially isolated, retaining their ultrastructural properties, and employed for further experiments.

10.1128/mSphere.01065-20.4 FIG S4 (A) Immunofluorescence analysis of E. coli BL21 (DE3) pRIL cells carrying plasmid pARR10 before or after induction for 16 h and partially purified inclusion bodies of CdeC. An antibody against 6×His was used as the first antibody, while the secondary antibody was conjugated with the Alexa Fluor 488 fluorophore. Inclusion bodies of CdeC from strain R20291 were purified as described in Materials and Methods. The partially purified inclusion bodies were adjusted to optical densities of 0.2 at 600 nm for immunofluorescence analysis using an anti-6×His antibody and a secondary antibody conjugated to the Alexa Fluor 488 fluorophore. (B) After purification, one sample of the CdeC bodies was treated for TEM. (Top) Selected individual inclusion bodies. (Bottom) Magnification of the thin selection. Download FIG S4, PDF file, 0.8 MB. Copyright © 2020 Romero-Rodríguez et al. 2020 Romero-Rodríguez et al. This content is distributed under the terms of the Creative Commons Attribution 4.0 International license.

A redox state is the ratio of the interconvertible oxidized and reduced forms of a specific redox couple (26). The redox state is important, since disulfide bond formation involves a reaction between the sulfhydryl (-SH) side chains of two cysteine residues that involves a nucleophilic attack of the S^−^ anion from one sulfhydryl group to a second cysteine, creating the disulfide bond. An oxidative environment will lead to oxidation of sulfhydryl groups and the formation of a cysteine bridge, while a reducing environment will generate the disruption of cysteine bridges (27). Since we observed that an oxidative environment affected the formation of the lamella-like structures upon heterologous expression in E. coli SHuffle T7, we asked whether increasing the reducing environment would affect the stability of the lamella-like structures.

**FIG 5:**
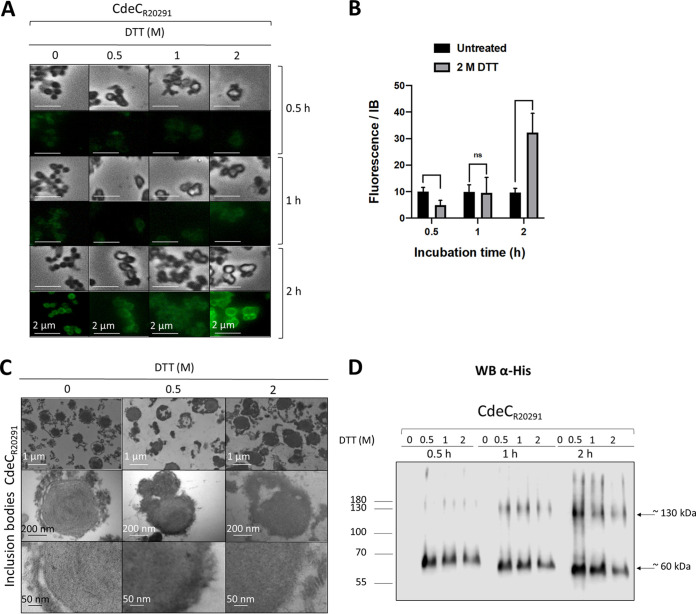
Effect of DTT treatment on the ultrastructure of the inclusion bodies of CdeC. (A) The partially purified inclusion bodies were treated with 0.5, 1, or 2 M DTT for 30, 60, or 120 min at 37°C. After each treatment, the pellet obtained was analyzed by immunofluorescence using anti-6×His as first antibody and a secondary antibody conjugated with Alexa Fluor 488. (B) Quantification of the fluorescence intensity of the inclusion bodies treated with 2 M DTT. The bars in the graph show the means ± standard errors of the means (SEMs) of 10 analyzed inclusion bodies. The statistical significance was determined by a two-way analysis of variance (ANOVA) using Sidak’s multiple-comparison test. ns, no significance. (C) Samples of purified inclusion bodies treated with 0.5 or 2 M DTT for 120 min were analyzed by TEM. (Top) Representative micrographs of several inclusion bodies. (Bottom) Magnified view of the thin section of inclusion bodies. (D) Western blot analysis of CdeC inclusion bodies after DTT treatment. The resulting final pellets of each treatment were electrophoresed in 12% SDS-PAGE gels, and His-tagged immunoreactive proteins were detected by Western blotting as described in Materials and Methods. Molecular mass (kDa) markers are indicated at the left, and molecular mass of the detected His-tagged immunoreactive bands is indicated at the right.

Therefore, we selected the reducing agent DTT, which maintains sulfhydryl (-SH) groups in a reduced state; it is effective for reducing the disulfide bridges in proteins and the cross-linker *N*,*N*′-bis(acryloyl)cystamine. Phase-contrast micrographs demonstrate that the inclusion bodies retained their phase-bright properties upon treatment with various DTT concentrations (Fig. 5A). The DTT treatment also resulted in the transition of the inclusion bodies from spherical forms to brighter amorphous structures (Fig. 5A), suggesting a disruption of the inclusion bodies’ compact structure. To assess if the amorphous structures generated after the DTT treatment resulted in less compact structures and, therefore, greater accessibilty to the anti-His antibody, we performed an immunofluorescence assay of each DTT treatment. The immunofluorescence of the partially purified inclusion bodies revealed a qualitative increase in the fluorescence intensity as the concentration of DTT and the incubation time increased. The highest intensity of fluorescence was observed when incubating inclusion bodies with 2 M DTT for 2 h (Fig. 5A). The quantification of the fluorescence intensity of inclusion bodies treated with 0.5, 1, and 2 M DTT for 2 h showed that the fluorescence intensity of inclusion bodies increased 3-fold when treated with 2 M DTT compared to that of inclusion bodies not treated with DTT. In contrast, the fluorescence intensities obtained upon treatment of inclusion bodies with 0.5 and 1 M DTT were lower than those of inclusion bodies not treated with DTT (Fig. 5B). Overall, these observations suggest that the His tag is less accessible when CdeC is present in lamella-like structures.

Next, to investigate the effect of DTT treatment in the inclusion bodies’ ultrastructure, samples treated with 0.5 and 2 M DTT for 2 h were observed by TEM. As seen, the characteristic ultrastructure, organized lamella like, was mostly lost after the treatment with DTT (Fig. 5C). We also observed that some filamentous fragments of the inclusion bodies were lost after the DTT treatment, suggesting those pieces correspond to CdeC aggregates or multimers. Therefore, to understand DTT’s effects on the CdeC inclusion bodies, DTT-treated samples were also resolved by SDS-PAGE prior to immunoblotting with anti-His antibodies. The treatment resulted in the appearance of immunoreactive bands, probably due to the release of monomeric and oligomeric forms of CdeC (Fig. 5D). Treatment of the inclusion bodies with DTT for 30 min resulted in the emergence of a protein band of approximately 60 kDa, suggesting a monomeric form of CdeC. Nevertheless, after 60 min of incubation with DTT, a multimeric form with a molecular weight of approximately 120 kDa was observed by Western blot analysis. These higher-molecular-weight bands may correspond to CdeC dimers or trimers. Interestingly, the longer the incubation time, the further the migration of immunoreactive bands corresponding to the putative monomeric form and closest to the 55-kDa weight (Fig. 5D). Overall, these results indicate that DTT treatment resulted in the abolition of lamella-like structures and allowed the release of monomeric and multimeric forms of CdeC from the inclusion bodies into the supernatants.

### CdeC redox‐sensitive disordered and residue-contact regions.

**FIG 6:**
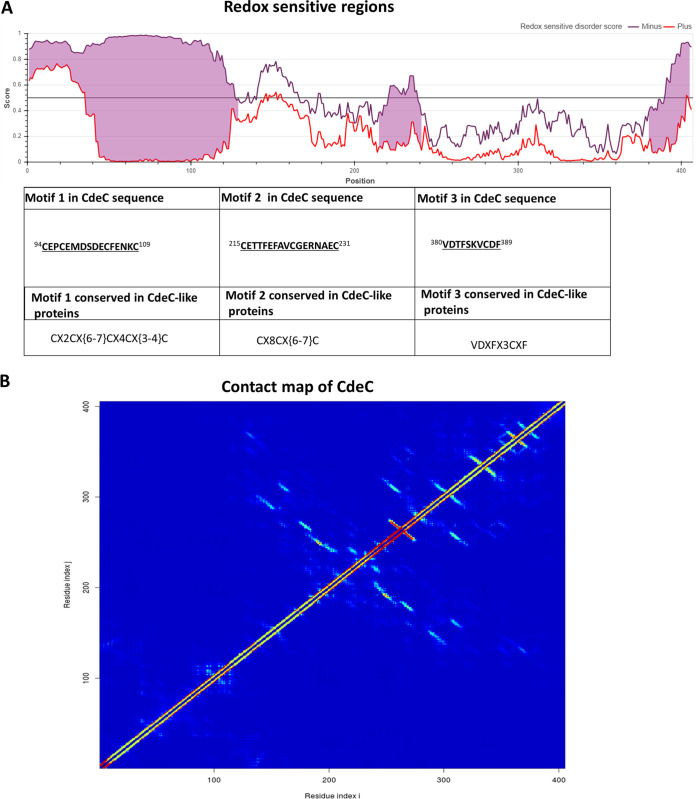
Redox‐sensitive disordered regions and residue contact map of CdeC. (A) Schematic representation of the prediction of redox-sensitive disordered regions, where the *x* axis represents the probability that one position of the protein is disordered (1) or ordered (0). The violet and red lines represent the state achieved through cysteine stabilization (redox plus) and without cysteine stabilization (redox minus), respectively. When these two profiles disagree, a redox-sensitive region is highlighted in pink. The table presents information about the amino acid sequence of the regions and the motifs involved. The motifs are in boldface and underlined in the amino acid sequence. The superscript numbers show the amino acid position in the CdeC protein of the C. difficile R20291 strain. (B) Contact map of CdeC from C. difficile R20291. A heat map is plotted to show the contact map of interacting inter-residues of CdeC inferred from the protein’s amino acid sequence. The heat map pattern ranges from red (100% chance of contact) to blue (0% chance of contact). The prediction was made in the web server DeepMetaPSICOV 1.0 (23).

As described above, CdeC self-assembly properties involved in forming the lamella-like structures depend, at least in part, on the formation of disulfide bridges. However, since CdeC contains 37 cysteine residues in its primary sequence, which might be interacting to drive the formation of the lamella-like structures, we sought to refine these regions by identifying the redox-sensitive disordered regions (RSDRs) using IUPred2A (28). Disordered proteins can mediate protein-protein interactions by recognizing specific partners and undergo a disorder-to-order transition by adopting a more structured conformation (29). The transition can be induced by interactions with other macromolecules or changes in environmental factors, such as pH, temperature, or redox potential (28). The critical sensors built into these redox-regulated proteins are cysteine residues, which can undergo reversible thiol oxidation in response to the oxidation status of the molecular environment (30). Under reducing conditions, cysteine residues can behave as polar amino acids, most similarly to serine, without contributing much to protein stability (28). However, they can also play essential roles in stabilizing the folded conformation by coordinating Zn^2+^ ions under reducing conditions or by forming disulfide bonds commonly used by extracellular proteins that experience oxidative conditions (28). Using C. difficile R20291 CdeC protein, redox-sensitive disordered regions were evaluated (Fig. 6A). We identified the probability of specific regions being in a disordered or an ordered conformation given its redox state. As seen in Fig. 6A, three regions on the protein may have conformations sensitive or dependent on the redox state. The first redox-sensitive region is harbored by residues 94 to 109 (CEPCEMDSDECFENKC), which has a predicted CX2CX(6-7)CX4CX(3-4)C sequence motif. The second redox-sensitive region is harbored by residues 215 to 231 (CETTFEFAVCGERNAEC), which exhibits a predicted CX8CX(6-7)C sequence motif. Moreover, the third redox-sensitive region (VDTFSKVCDF) spans from residues 380 to 389 and exhibits a VDXFX(3)CXF sequence-motif.

To further analyze the putative relevance of the predicted redox-sensitive motifs and to identify possible pairs of interacting residues, a contact map (a graphic description of interacting partners) was generated using the webserver DeepMetaPSICOV 1.0 (31). A color scale based on 100% contact probability (red) to 0% (blue) shows the matrix representation of possible contacts (Fig. 6B). There is a hot-spot region, between positions 200 to 250, enriched with putative interacting residues.

### Effect of domain deletions of CdeC on oligomerization and self-assembly properties.

**FIG 7:**
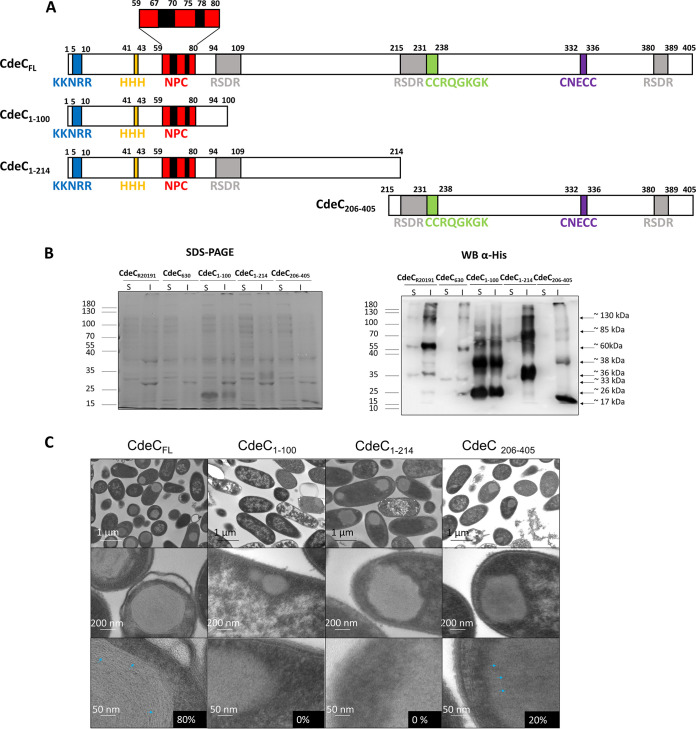
Construction and analysis of CdeC truncations. (A) Schematic representation showing the sequences of CdeC (CdeC_FL_) and the three variants (CdeC_1–100_, CdeC_1–214_, and CdeC_206–405_). (B) Recombinant proteins were expressed in E. coli BL21(DE3) pRIL carrying plasmids pARR19, pARR20, pARR7, and pARST1, respectively, induced with 0.5 mM IPTG for 16 h at 37°C. The cells were disrupted in soluble and insoluble lysis buffers and electrophoresed in 15% SDS-PAGE gels; His-tagged immunoreactive proteins were detected by Western blotting as described in Materials and Methods. Each lane was loaded with 2 μg of protein lysate. Molecular mass (kDa) markers are indicated at the left, and molecular mass of the detected His-tagged immunoreactive bands is indicated at the right. (C) Thin sections of E. coli BL21(DE3) pRIL expressing CdeC from C. difficile R20291 and its truncated forms. The sections were analyzed by transmission electron microscopy, as described in Materials and Methods. (Top) Representative micrographs of several E. coli cells are shown. (Bottom) Magnified views of the thin section of inclusion bodies inside E. coli BL21(DE3) pRIL. Blue arrowheads indicate the presence of lamella-like structures. Numbers on black boxes show the percentages of cells with IBs with lamella-like structures.

The self-assembly of short peptides has been actively studied in recent years (32). The process is generally driven by specific, spontaneous, and noncovalent chemical interactions. In the case of CdeC, some regions of the protein might have the auto-assembly capacity. Since the redox environment and DTT treatment affect the organized ultrastructure of the inclusion bodies, these organizations could depend, at least in part, on the formation of disulfide bridges, suggesting that cysteine residues could be implicated in self-assembly properties. As mentioned before, the sequence of CdeC contains several cysteine residues and sequence repeats (Fig. 7A), but the relevance of these residues is unknown and may be implicated in the organization of lamella-like structures of CdeC.

Therefore, to define if some of the sequence repeats or some regions in CdeC are prone to aggregation, three different variants were constructed. The first variant (CdeC_1–100_) includes the first methionine to the aspartate in position 100. Special features included in this variant are the sequence motifs KKNKRR, HHH, and NPC from the N terminus of the sequence of CdeC but lacks a full RSDR to evaluate the effect of the multiple NPC repeats. (Fig. 7A). The predicted molecular weight of this variant is 11.6 kDa. The second variant, CdeC_1–214_, goes from the initial methionine to the asparagine in position 214. This variant includes residues 1 to 100 the of N terminus of CdeC, the KKNKRR, HHH, and NPC sequence motifs, and the central region of the sequence of CdeC (Fig. 7A). The redox-sensitive disordered region (RSDR) CEPCEMDSDECFENKC is also present in this variant (Fig. 7A). For this variant, the predicted molecular weight is 22 kDa. The third variant, CdeC_206–405_, starts with the proline in the 206th position and ends with the arginine at 405, the last amino acid of the CdeC primary sequence. This variant includes the CCRQGKGK and CNECC sequence motifs present in the C-terminal domain of CdeC (Fig. 7A) and two redox-sensitive disordered regions, CETTFEFAVCGERNAEC and VDTFSKVCDF (Fig. 7A). The predicted molecular weight of this variant is 23.9 kDa. These three CdeC variants were expressed in E. coli BL21(DE3) pRIL, and E. coli lysates obtained were analyzed by SDS-PAGE and Western blotting (Fig. 7B). Western blot analysis showed the presence of multimeric forms of CdeC_1–100_, CdeC_1–214_, and CdeC_206–405_ variants. For variant CdeC_1–100_, Western blot analysis revealed at least three multimeric forms of apparent molecular weights of 26, 38, and 60 kDa in the soluble and insoluble fractions, suggesting the formation of multimeric forms, probably dimers or trimers, of CdeC_1–100._ For the CdeC_1–214_ variant, Western blot analysis of soluble and insoluble fractions showed the multimeric forms with molecular weights of approximately 33 (putative monomer) and 70 (putative dimer) kDa in the soluble and insoluble fractions obtained from the E. coli lysates. Besides, a multimeric form with an apparent molecular weight of 120 kDa was observed in the insoluble fraction that was immunoreactive with the anti-His antibody. For variant CdeC_206–405_, Western blot analysis revealed at least four multimeric forms with molecular weights of approximately 17, 38, 60, and 85 kDa in the insoluble fraction. Presumably, the band with an apparent molecular weight of 17 kDa could be the monomeric form of CdeC_206–405_ (Fig. 7A). The higher-molecular-weight bands may be multimeric forms of CdeC, including dimers, trimers, and tetramers.

Next, to assess the aggregation and self-organization capacity of the different CdeC variants, E. coli cells overexpressing these variants were analyzed by TEM. TEM of the overexpression in E. coli BL21(DE3) pRIL of the three variants (CdeC_1–100_, CdeC_1–214_, and CdeC_206–405_) showed that CdeC_1–214_ and CdeC_206–405_ variants were aggregated in inclusion bodies of similar sizes (Fig. 7C). These results agree with the Western blot analysis, where most of the protein was found in the insoluble fraction. In contrast, the CdeC_1–100_ variant did not appear to form inclusion bodies, and it was found, qualitatively, in the same amounts in soluble and insoluble fractions (Fig. 7B). Furthermore, the inclusion bodies of variants CdeC_1–214_ and CdeC_206–405_ are likely as big as those of the full-length protein (CdeC_FL_) but lack the lamella-like structure (Fig. 7C). For the CdeC_206–405_ variant, in 20% of the analyzed cells, the inclusion bodies appeared to contain discrete laminations (Fig. 7C), but not as clearly as for CdeC_FL,_ where 80% of the IBs displayed clear lamella-like structures. It would be worth studying in detail if this region has a significant role in CdeC organization. Overall, these results demonstrate that the different oligomerization properties of CdeC may span the entire amino acid sequence. However, additional information is needed to address if the self-assembly properties of CdeC are directed by specific domains or regions of the protein.

## DISCUSSION

The outermost layer of C. difficile spores is critical for understanding CDI’s pathogenesis and for developing novel intervention therapies. Recent work has shown that this outer layer’s composition includes three cysteine-rich proteins (i.e., CdeA, CdeC, and CdeM) (8, 11). Genetic studies of CdeC and CdeM have shown that although both are essential for the assembly of the exosporium layer, CdeC has pleiotropic roles in the biology of C. difficile spores, including spore coat assembly, spore resistance, and spore germination (8, 21). However, the underlying mechanism that drives CdeC- and/or CdeM-mediated assembly of the outermost exosporium layer remains unclear. In this work, we began dissecting the underlying molecular basis that drives exosporium assembly of C. difficile spores by demonstrating that although CdeA, CdeC, and CdeM oligomerize upon heterologous expression in E. coli, only CdeC exhibits self-assembly properties that lead to the formation of inclusion bodies with lamella-like structures.

The first major finding of this work was that the cysteine-rich proteins present in the exosporium of C. difficile, CdeA, CdeC, and CdeM, can form multimers (Fig. 1A). Upon heterologous expression in E. coli, all three proteins (i.e., CdeA, CdeC, and CdeM) are detected not only as monomers but also as higher-molecular-weight immunoreactive species that are stable under denaturation conditions (Fig. 1A), mostly in the insoluble fraction. The immunoreactive bands always ran slower than expected. This shift in migration of the cysteine-rich proteins could be attributed to the formation of strong bonds that are stable under denaturing conditions or, alternatively, to the presence of traces of a strong prooxidant (ammonium persulfate) during SDS-PAGE and the absence of disulfide reducing agents, which are present in the loading buffer but are not able to migrate during electrophoresis, thus allowing the reoxidation of cysteine residues and formation of disulfide bonds as suggested by Achilli et al. (33). Interestingly, the formation of lamella-like structures and the presence of oligomers of CdeC appeared not to be affected by temperature (see Fig. S1 in the supplemental material). The multimeric forms have also been observed in cysteine-rich proteins of the exosporium layer of spores of the Bacillus anthracis*/cereus/thuringiensis* group, such as ExsY (14); similarly, cysteine-rich proteins of the crust layer of B. subtilis spores, such as CotY, also have been observed to form stable oligomers (12). Two oligomeric forms of 19.4 and ∼120 kDa were observed for ExsY protein (14), while a high molecular weight oligomer >80 kDa was observed was observed for the CotY protein (34). Both ExsY and CotY oligomers, when treated with a reducing agent such as DTT and heat, are disassembled into their monomeric forms (12, 14). The formation of CotY oligomers was previously reported when analyzing spore extracts by Western blotting. The presence of multimeric forms of 26, 56, and 78 kDa corresponding to monomer, dimers, and trimers, respectively, has been described (35). Notably, the recombinant proteins ExsY and CotY produced in E. coli self-assembled, forming hexameric crystal structures that were characterized by heat stability and endurance against denaturing chemicals such as SDS. The total disruption of those structures was accomplished only when heat and a reducing agent (DTT) were applied (12, 14). In Clostridium sporogenes, it was also observed that the cysteine-rich exosporium protein, CsxA, self-assembled into a highly thermally stable structure identical to that of the native exosporium when expressed in E. coli (36). Therefore, it is tempting to propose that cysteine-rich proteins drive the exosporium assembly by building a self-assembled structure that serves as a scaffold for the latter recruitment of other exosporium constituents, and this mechanism may be conserved in the *Clostridiales*.

A second major contribution of this work is the observation of organized inclusion bodies upon CdeC overexpression. Transmission electron micrographs showed that inclusion bodies of CdeC exhibited an organized lamella-like structure (Fig. 2C). Interestingly, we recently demonstrated that overexpression of CdeC in C. difficile R20291 resulted in the formation of an aberrant exosporium with a visible disorganized lamella-like structure (37), suggesting that these lamella-like structures are due to CdeC. In many cases, assembly is primarily driven by hydrophobic interactions, attenuated by electrostatics with directional specificity imposed by electrostatic, van der Waals, and hydrogen bonding interactions (38). Although the precise mechanism that guides the formation of the lamella-like is unknown, the bioinformatic analysis predicted three conserved redox-sensitive disordered regions (RSDRs) (Fig. 6) whose role in CdeC organization is worth analyzing. We looked for those RSDRs in the ExsY and CotY proteins, since these are prototypical self-assembled proteins. While no conserved RSDR sequences were found in ExsY or CotY, conserved RSDRs were found in CdeC-like proteins from the *Peptostreptococcaceae* family (*Clostridium* cluster XI) of spore formers (Fig. 6 and Table S4).

TEM analysis as the CdeC variants showed that the proteins’ central and carboxyl regions tend to form aggregates but cannot form the lamella-like structure like the full-length CdeC (Fig. 7). Interestingly, in around 20% of the IBs from the variant CdeC_206-405_ (C-terminal variant), faint lamella-like structures were observed (Fig. 7). The use of more sensitive technology, such as cryo-electron microscopy (cryoEM), would help in understanding the structural features of CdeC. One constraint in our study is the utilization of truncated variants of the proteins, since this approximation may impair the correct folding of the protein. There is high homogeneity of CdeCs across C. difficile strains, as evidenced by our bioinformatics analysis of C. difficile published genomes, where the lowest identity at the amino acid level was greater than 90%. Interestingly, despite the differences at the amino acid level between CdeCs of strains R20291 and 630, no differences in the formation of lamella-like structures were evidenced (Fig. 3), indicating that CdeC’s self-assembly properties are conserved across C. difficile strains. It would be interesting to trace the evolution of self-assembled properties of CdeC and answer the question if the most recent ancestor of the CdeC self-organized in these lamella-like structures.

A major question raised by this work is how does the redox environment affect the formation and stability of the lamella-like structures in CdeCs inclusion bodies? Disulfide bridge formation requires an oxidative environment for proper formation; thus, it was somewhat surprising that CdeC expression in SHuffle T7, with a highly oxidative cytoplasmic environment, leads to inclusion bodies that lacked the formation of the lamella-like structures (Fig. 4). A plausible explanation could be the aberrant formation of disulfide bridges within and/or between redox-sensitive regions under a highly oxidative environment. That is, multiple nonspecific cysteine bridges formed due to the high number of consecutive cysteines in the CdeC sequence might not be appropriately isomerized by the DsbC chaperone, leading to a protein that is improperly folded and therefore unable to form native disulfide bonds with itself or another monomer correctly. Another explanation that could not be ruled out is the lower expression of CdeC in the SHuffle T7 strain (Fig. 4), suggesting that the organization in lamella-like structures of CdeC may respond to a critical concentration of the protein. However, aggregates were visualized by TEM, and high-molecular-weight immunoreactive bands were also detectable. These results suggest that strong interactions stabilize CdeC assembly in addition to disulfide bridges, a property that has also been observed for the B. subtilis crust protein CotY, where strong denaturing conditions were not able to disrupt CotY crystals (12). These results are the first report of the ultrastructure of inclusion bodies formed by cysteine-rich proteins present in the exosporium of C. difficile; however, whether this organization is relevant for exosporium assembly remains unclear and is a matter of current work in our group.

## MATERIALS AND METHODS

### Bacterial strains, plasmids, and media.

Escherichia coli BL21(DE3) pRIL and SHuffle T7 (New England Biolabs, USA) strains were routinely grown in Luria-Bertani (LB) broth. The expression of recombinant proteins in E. coli was performed in LB broth supplemented with 0.5% glucose (LBG) (21 or 37°C at 2 × *g*). A detailed description of the genetic background and relevant characteristics of each strain are provided in Table S1 in the supplemental material.

10.1128/mSphere.01065-20.5 TABLE S1 Plasmids and strains used in this work. Download Table S1, DOCX file, 0.1 MB. Copyright © 2020 Romero-Rodríguez et al. 2020 Romero-Rodríguez et al. This content is distributed under the terms of the Creative Commons Attribution 4.0 International license.

### Construction of overexpression plasmids.

Phenol-chloroform-extracted genomic DNA from C. difficile R20291 (accession no. FN545816.1) strain was used as a template for the amplification of *cdeC* (CD0926), *cdeM* (CD1478), and *cdeA* (CD2262) genes and the truncated variants of *cdeC*. All amplifications were carried out with Phusion hot start high-fidelity DNA polymerase (Thermo Scientific, USA). The resulting amplicons were cloned into plasmid pET22b and (or) pETM11. CdeC was cloned by the traditional restriction ligation method in the pETM11 vector between NcoI and XhoI restriction sites, while the construction in pET22b was between the NdeI and XhoI sites. The truncated version of CdeC was cloned into pET22b, as described before. CdeM and CdeA were cloned into pETM11 by Gibson assembly between NcoI and XhoI sites (Gibson assembly cloning kit; New England Biolabs). All primers are listed in Table S2. The resulting plasmids are shown in Table S1. All the constructs were verified by DNA digestion and Sanger sequencing.

10.1128/mSphere.01065-20.6 TABLE S2 List of primers used in this study. Download Table S2, PDF file, 0.1 MB. Copyright © 2020 Romero-Rodríguez et al. 2020 Romero-Rodríguez et al. This content is distributed under the terms of the Creative Commons Attribution 4.0 International license.

### Overexpression of CdeA, CdeC, CdeM, and truncated variants of CdeC-6×His fusions.

Plasmids pDP339, pARR10 or pARR19, pARR2, pARR22, and pARR20, pARR7, and pARST1 containing *cdeC* (C. difficile strain 630, CD1067), *cdeC* (C. difficile strain R20291, CD0926), *cdeM*, *cdeA*, and the *cdeC* variants *cdeC_1–100_*, *cdeC_1–214_*, and *cdeC_206–405_*, respectively, were transformed into Escherichia coli strain BL21(DE3) pRIL. Also, plasmid pARR19 was transformed into E. coli strain SHuffle T7 (39). Transformed cells were used to inoculate 5 ml LBG medium (containing 50 μg ml^−1^ or 150 μg ml^−1^ kanamycin) and were grown overnight at 37°C and 2 × *g*. The overnight cultures (5 ml) were used to inoculate 600 ml of production medium. Cultures were grown to optical densities of between 0.7 and 0.9 at 600 nm, after which the temperature was maintained at 37°C or reduced to 21°C. The cultures were induced with 0.5 mM isopropyl-β-d-thiogalactoside (IPTG). Incubation was continued for 16 h and at 37°C and 2 × *g*; the cells were harvested by centrifugation at 5,853 × *g* for 15 min. The cell pellets were stored at −80°C.

Next, cells were first resuspended in 500 μl of soluble lysis buffer, 20 mM Tris-HCl (pH 7.8), 500 mM NaCl, 5 mM imidazole, 0.5% to 0.8% Triton X-100 and 0.1 mM protease inhibitors (Thermo Fisher, USA). Resuspended cultures were sonicated at the output of 12 W for six bursts of 15 s, separated by 15 s of cooling on ice-cold water. Lysates were centrifuged at 18,625 × *g* for 10 min, and the supernatant was saved as the soluble fraction; the pellet was subsequently resuspended in 500 μl of insoluble lysis buffer, 20 mM Tris-HCl (pH 7.8), 500 mM NaCl, 5 mM imidazole, and 8 M urea. Resuspended lysates were sonicated at the output of 21 W for six bursts of 30 s, separated by 30 s of cooling on ice-cold water, and saved as the insoluble fraction. Soluble and insoluble fractions were further analyzed by SDS-PAGE and Western blotting, as described below.

### SDS-PAGE and Western blot analysis of recombinant protein CdeC, CdeM, and CdeA and truncated variants of CdeC.

Fraction soluble and insoluble of recombinant CdeC, CdeM, and CdeA were mixed with 2× SDS-PAGE loading buffer and then were boiled for 5 min; 2 μg of total protein was loaded per lane. Samples were electrophoresed in SDS-PAGE gels (12% or 15% acrylamide). Some gels were directly stained with Coomassie brilliant blue, and in others, proteins were transferred to nitrocellulose membranes (Bio-Rad, USA) and blocked 1 h at room temperature with 5% skim milk in Tween-Tris-buffered saline (TTBS) (pH 7.4). The blocked membranes were probed overnight at 4°C with a 1:5,000 dilution of mouse anti-6×His antibodies (Thermo or Rockland Immunochemicals Inc., USA), rinsed three times with TBS-0.1% (vol/vol) Tween 20, and incubated for 1 h at room temperature with a 1:10,000 dilution of horseradish peroxidase (HRP)-conjugated anti-mouse IgG (Rockland Immunochemicals Inc.). Both primary and secondary antibodies were incubated in the presence of 5% milk (Sigma-Aldrich). HRP activity was detected with a chemiluminescence detection system (Li-COR imaging system) using the Clarity chemiluminescent detection system HRP substrate (Bio-Rad). Each Western blot also included 3 μl of a Page Ruler Plus prestained protein ladder (Thermo). The images shown in the figures are representatives of at least three independent experiments.

### Purification of inclusion bodies.

Recombinant CdeC was expressed in E. coli BL21(DE3) pRIL according to the following conditions: the expression was induced with 0.5 mM IPTG for 16 h at 37°C and agitation at 2 × *g* in LBG medium. Cells were concentrated by centrifugation at 5,853 × *g* for 15 min at 4°C, and the inclusion bodies were isolated as described below. The pellet was resuspended with 30 to 35 ml of 10 mM EDTA in 1× phosphate-buffered saline with Tween 20 (PBST) per liter of overexpression culture. The cells were then lysed with 10 mg/ml lysozyme for 1 h at 37°C. After that, cells were sonicated at 12 W for 15 s on ice and centrifuged at 5,853 × *g* for 45 min at 4°C. The supernatant obtained was discarded, and the pellet was washed two times with 2% Triton X-100 in 1× PBS by centrifugation at 5,853 × *g* for 20 min at 4°C. The cell lysates were sonicated at a power of 12 W for 15 s on ice and passed through a 45% Nicodenz gradient for 50 min at 5,853 × *g* at 4°C to separate the cells and inclusion bodies. Finally, the inclusion bodies were washed twice with 0.1 mM phenylmethylsulfonyl fluoride (PMSF) in 1× PBS by centrifugation at 5,853 × *g* for 20 min, adjusted to an optical density of 0.2 at 600 nm in a volume of 20 μl, and stored at −80°C. The images shown in the figures are representatives of at least three independent experiments.

### Bioinformatic analysis.

One thousand eight hundred thirty-five publicly available C. difficile genomes were downloaded from GenBank, and their MLSTs and clades were determined in the PubMLST database (see Table S3). The amino acid sequence of CdeC was searched using tBLASTn v 2.9.0+. The nucleotide sequences of the coding DNA sequences (CDSs) corresponding to the *cdeC* gene were extracted using threshold hits with at least 90% coverage and 30% identity concerning the reference gene of the C. difficile 630 strain, leaving all parameters set at the default. The genes found are listed in Table S4. To make a rooted phylogenetic tree explaining the evolutionary history of CdeC, the NCBI RefSeq database was searched using the same strategy, leaving out all hits with organisms that were described as C. difficile (taxonomy identifier [tax id] 1496) and that had a coverage of less than 90% and 30% identity to the reference gene of the C. difficile 630 strain. The genes found and the species to which they belong are listed in Table S4. The files were concatenated and clustered into single alleles using CD-HIT-EST v4.8.1. The single alleles are listed in Table S5. These single alleles were aligned using the AlignTranslation function of the R DECIPHER package v2.16.1 with the default parameters. The multiple sequence alignment can be found in Table S6 as nucleotide or amino acid sequences. To perform the phylogenetic inference, the GTR+I+G substitution model was determined by the Akaike information criterion (AIC) and Bayesian information criterion (BIC) methods using the jModelTest v2.1.10 program on nucleotide alignment (Table S6). Phylogenetic inference was performed using the Bayesian method with the program BEAST v1.10.4, using for this purpose 6 independent chains of 10,000,000 states sampling every 1,000 states. Valid sample size values were >200 for all parameters, and convergence and mixing were assessed using Tracer v1.7.1. Log and tree files were summarized with tree annotator v2.4.8 using 10% burning, along with maximum clade credibility and node heights at the median. To complement the phylogenetic inference, a second inference was made using the maximum likelihood method with the RAxML v8.2.12 program, indicating that the outgroup taxa of the analysis are from the sequences that do not belong to the C. difficile species. The topologies of the two trees were compared using the web service http://phylo.io/, and the phylogenetic trees were visualized at https://itol.embl.de/. Also, because cysteines are amino acids that can command the transition from disorder to order due to potential redox changes in the environment, redox-sensitive disordered regions were evaluated on all single-allele proteins using a webserver (28). To predict the contact map of CdeC, we used the program DeepMetaPSICOV 1.0 (31) under default settings.

10.1128/mSphere.01065-20.7 TABLE S3 MLSTs and clades of 1,835 publicly available C. difficile genomes. Download Table S3, XLSX file, 0.1 MB. Copyright © 2020 Romero-Rodríguez et al. 2020 Romero-Rodríguez et al. This content is distributed under the terms of the Creative Commons Attribution 4.0 International license.

10.1128/mSphere.01065-20.8 TABLE S4 Descriptions and sequences of analyzed genes. Download Table S4, XLSX file, 0.1 MB. Copyright © 2020 Romero-Rodríguez et al. 2020 Romero-Rodríguez et al. This content is distributed under the terms of the Creative Commons Attribution 4.0 International license.

10.1128/mSphere.01065-20.9 TABLE S5 Accession codes and sequences of the unique alleles found in C. difficile genomes. Download Table S5, XLSX file, 0.1 MB. Copyright © 2020 Romero-Rodríguez et al. 2020 Romero-Rodríguez et al. This content is distributed under the terms of the Creative Commons Attribution 4.0 International license.

10.1128/mSphere.01065-20.10 TABLE S6 Multiple sequence alignment of unique alleles of *cdeC.* Download Table S6, XLSX file, 0.1 MB. Copyright © 2020 Romero-Rodríguez et al. 2020 Romero-Rodríguez et al. This content is distributed under the terms of the Creative Commons Attribution 4.0 International license.

### DTT treatment of CdeC inclusion bodies.

The isolated CdeC inclusion bodies were treated with different concentrations of dithiothreitol (DTT; Thermo Scientific, USA), a disulfide reducing agent, for different incubation times. CdeC inclusion bodies partially purified and adjusted to optical densities of 0.2 at 600 nm in a volume of 20 μl were treated with 500 mM, 1 M, and 2 M DTT during 30-, 60-, and 120-min incubations at 37°C. The effect of treatment of CdeC inclusion bodies with 2 M DTT during different incubation times was evaluated by SDS-PAGE, Western blotting, and immunofluorescence. The images shown in the figures are representatives of at least three independent experiments.

### Immunofluorescence of overexpressed recombinant cysteine-rich proteins and inclusion bodies of CdeC with or without DTT treatment.

Immunofluorescence analysis was performed to assess the overexpression of recombinant CdeC, CdeM, and CdeA proteins in E. coli and the partially purified CdeC inclusion bodies treated and not treated with DTT. For this purpose, recombinant CdeC, CdeM, and CdeA proteins were overexpressed in E. coli strain BL21(DE3) pRIL, as described above. An aliquot of 1 ml was taken from both IPTG-induced and non-IPTG-induced cultures and concentrated by centrifugation at 4,107 × *g* for 8 min at 4°C. Before the immunofluorescence analysis, E. coli cells were fixed in the LBG medium supplemented with 3% paraformaldehyde (PFA) and incubated for 10 min at room temperature and then for 30 min on ice. The E. coli cells were washed 3 times with 1× PBS by centrifugation at 4,107 × *g* for 8 min at 4°C and resuspended in GTE buffer (50 mM glucose, 10 mM EDTA, and 20 mM Tris, pH 7.5) supplemented with 0.7 mg/ml lysozyme. In the case of the partially purified CdeC inclusion bodies not treated with DTT, the immunofluorescence analysis was performed directly. In contrast, the inclusion bodies of CdeC were treated as described in “DTT treatment of CdeC inclusion bodies” and washed 3 times with 1× PBS by centrifugation 5,853 × *g* for 20 min at 4°C to remove excess DTT.

For immunofluorescence analysis, sample droplets of 2 μl were added to poly-l-lysine-pretreated coverslips and dried for 10 min at 37°C. The samples were fixed with 4% PFA for 15 min at room temperature, except for E. coli cells, which were previously fixed, and were washed three times with 1× PBS and once with Milli-Q H_2_O. The cells samples were blocked with 1% bovine serum albumin (BSA) in 1× PBS for 1 h at room temperature in a humid chamber. Then, the samples were incubated with anti-6×His mouse primary antibody (Thermo Scientific, USA) at a 1:500 dilution in 1× PBS with 1% BSA for 1 h at room temperature in a humid chamber, and 3 washes were performed with 1× PBS and one with Milli-Q H_2_O. The samples were incubated with Alexa Fluor 488 anti-mouse IgG secondary antibody (Abcam, UK) at a 1:400 dilution in 1× PBS with 1% BSA for 1 h at room temperature in a humid chamber in the dark. Later, the samples were washed three times with 1× PBS and once with Milli-Q H_2_O. Finally, the coverslips were dried for 10 min at 37°C, mounted with 3 μl of Dako reagent, dried for 30 min at 37°C, and stored at 4°C until observed under an Olympus BX53 fluorescence microscope. The images shown in the figures are representatives of at least three independent experiments.

### Transmission electronic microscopy.

Recombinant proteins CdeC, CdeM, and CdeA and truncated variants of CdeC were expressed in E. coli, as described above. The E. coli cells were concentrated by centrifugation at 5,853 × *g* for 15 min, fixed with 3% glutaraldehyde in 0.1 M cacodylate buffer (pH 7.2), incubated overnight at 4°C, and stained for 30 min with 1% tannic acid. Samples were further embedded in a Spurr resin (40). Thin sections of 90 nm were obtained with a microtome, placed on glow discharge carbon-coated grids, and double lead stained with 2% uranyl acetate and lead citrate. Spores were analyzed with a Philips Tecnai 12 Bio Twin microscope at Unidad de Microscopía Avanzada in Pontificia Universidad Católica de Chile. To analyze the thickness of the interspace between the lamination-like structures, we randomly selected 10 IBs per strain. Each individual IB was divided along its axial and longitudinal axes into four quadrants. Next, five measurements per quadrant were measured, giving a total of 20 measurements per IB. Therefore, 200 measurements for the lamination interspace were obtained per strain and plotted in a frequency distribution graph. The data were fit to a Gaussian curve using Graph Pad Prism 8 software.
