# Supplementary figures and images for: The Clostridioides difficile Cysteine-Rich Exosporium Morphogenetic Protein, CdeC, Exhibits Self-Assembly Properties That Lead to Organized Inclusion Bodies in Escherichia coli
Source: mSphere. 2020 Nov 18;5(6):e01065-20. doi: 10.1128/mSphere.01065-20 (PMC7677010; doi:10.1128/mSphere.01065-20)

**Fig. S1**

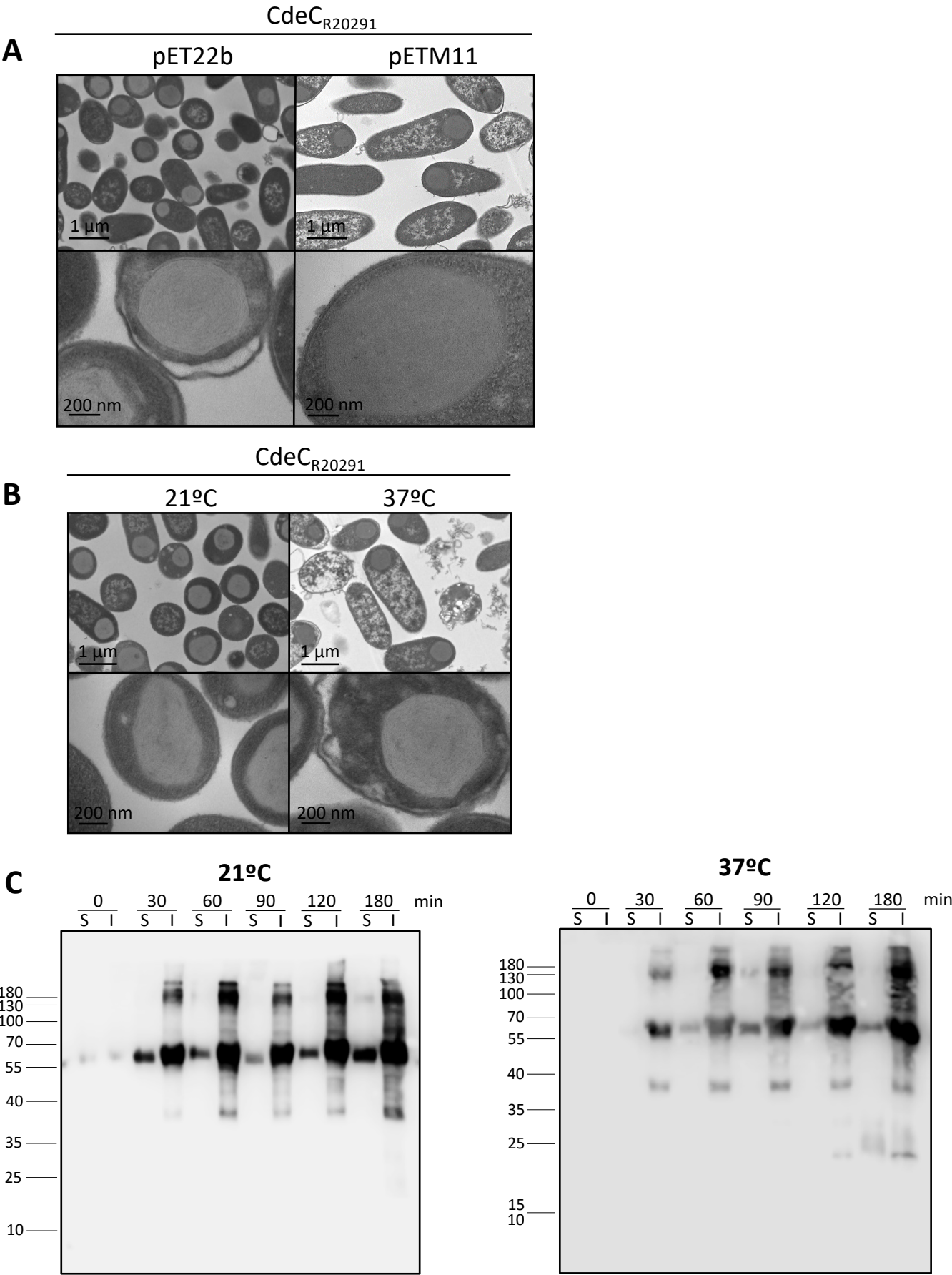

Supplement: FIG S1 [file mSphere.01065-20-sf001.pdf]

Fig. S3

A

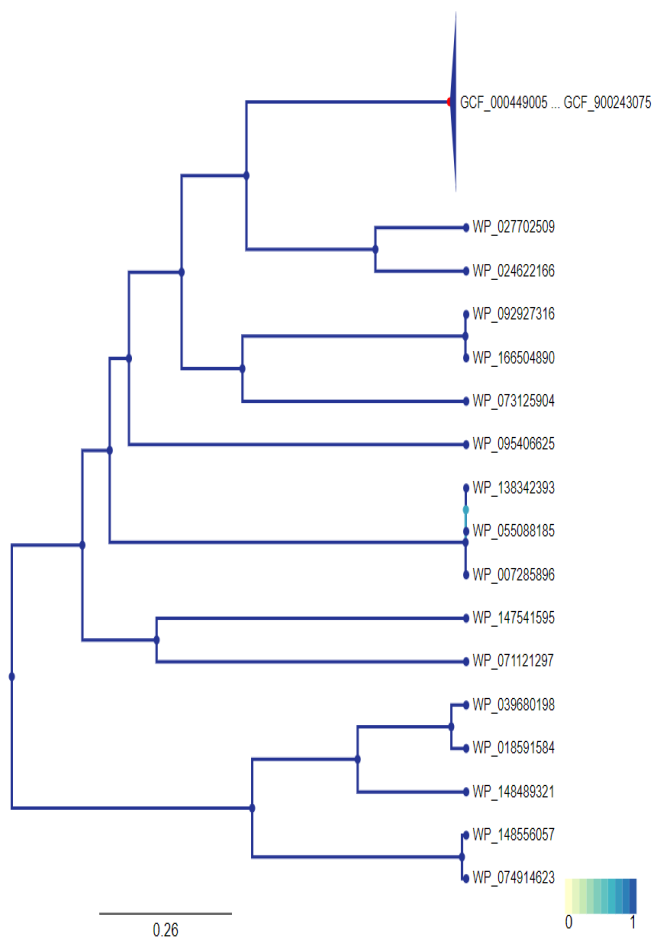

B

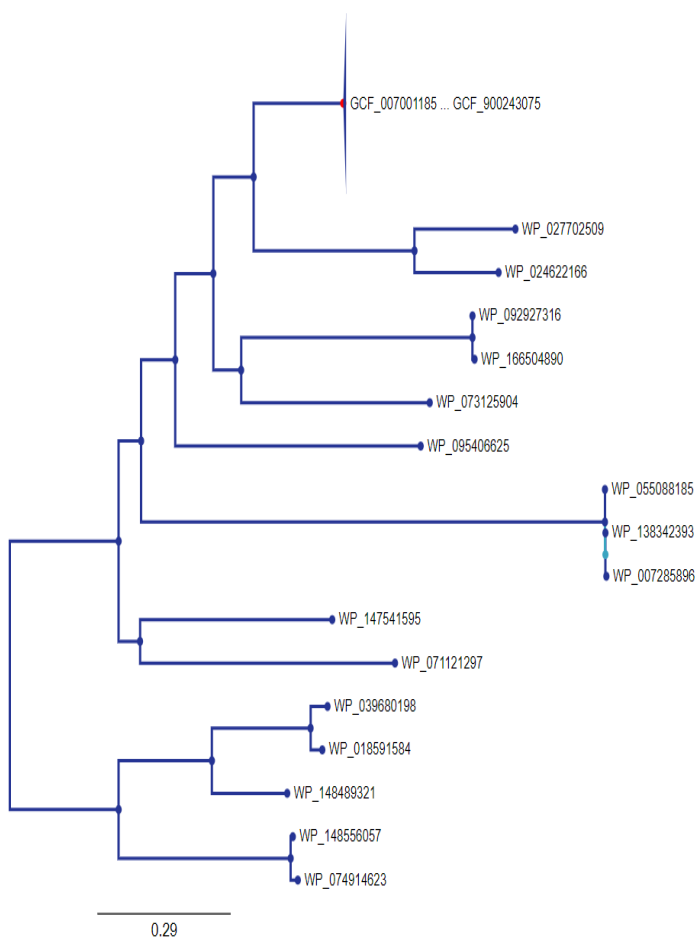

Supplement: FIG S3 [file mSphere.01065-20-sf003.pdf]

Fig. S4

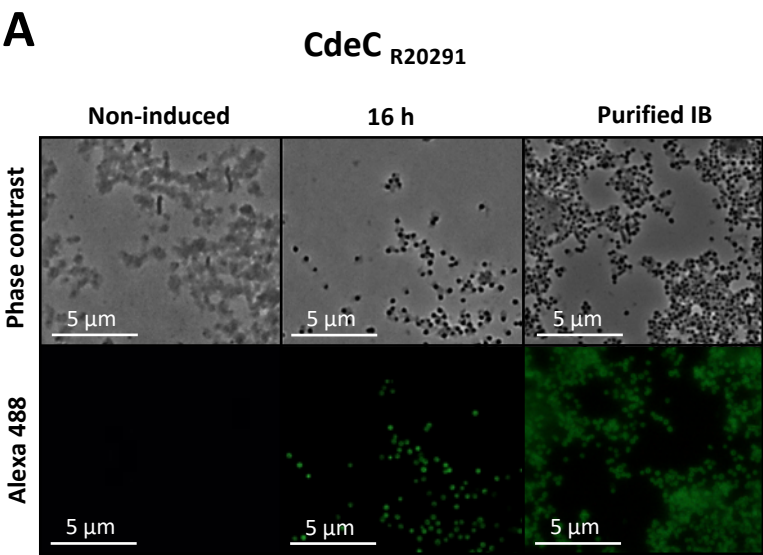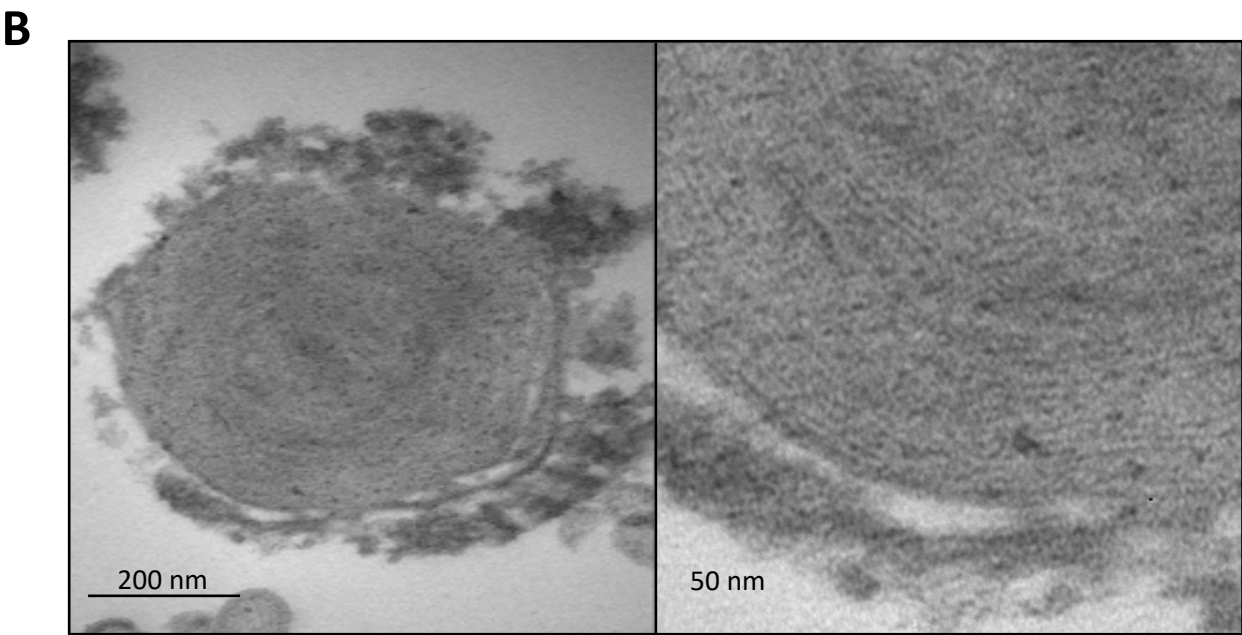

Supplement: FIG S4 [file mSphere.01065-20-sf004.pdf]
