# Supplementary material for: The Clostridioides difficile Cysteine-Rich Exosporium Morphogenetic Protein, CdeC, Exhibits Self-Assembly Properties That Lead to Organized Inclusion Bodies in Escherichia coli
Source: mSphere. 2020 Nov 18;5(6):e01065-20. doi: 10.1128/mSphere.01065-20 (PMC7677010; doi:10.1128/mSphere.01065-20)
Supplement: FIG S2 [file mSphere.01065-20-sf002.pdf]

A)

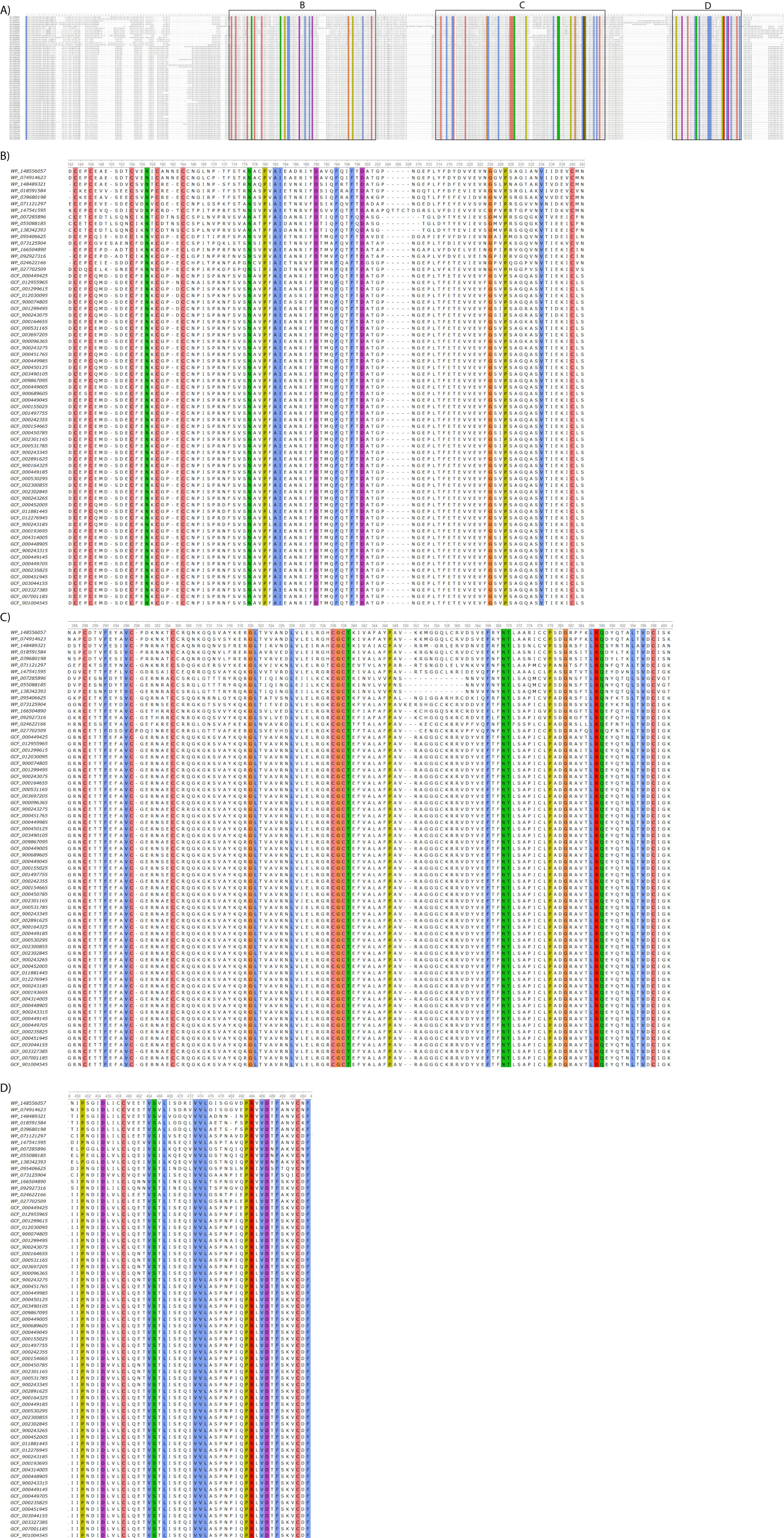

B)

C)

D)
