# Supplementary material for: The Clostridioides difficile Cysteine-Rich Exosporium Morphogenetic Protein, CdeC, Exhibits Self-Assembly Properties That Lead to Organized Inclusion Bodies in Escherichia coli
Source: mSphere. 2020 Nov 18;5(6):e01065-20. doi: 10.1128/mSphere.01065-20 (PMC7677010; doi:10.1128/mSphere.01065-20)
Supplement: TABLE S2 [file mSphere.01065-20-st002.pdf]

Table S2 List of primers used in this study

**Primer name**	**Primer sequence** [a] **5'-3´**	**Position** [b]	**Gene** [c]	**Use**
FP-cdeC-CD0926-N*co*I V2	CGACCATGGCCATGCAAGATTATAAAAAAAATAAAAGAAGAATG	+3 to +36	*cdeC*-CD0926	The forward primer used to clone *cdeC* of in the expression vector pETM11.
RP-CdeC-CD0926-X*ho*I V2	GCGACTCGAGTCTGTGGCAACTTGGCTTTCC	-1195 to-1215	*cdeC*-CD0926	The reverse primer used to clone *cdeC* in the expression vector pETM11.
FP-CD1067-N*de*I	GACCATATGCAAGATTATAAAAAAAAATAAAAGAAGAATGATGAATCAGC	+3 to +45	*cdeC*-CD0926	Forward primer used to clone *cdeC* in the expression vector pET22b.
RP-CD1067-X*ho*l	GACCTCGAGTCTGTGGCAACTTGGCTTTCCACTTC	-1189 to-1215	*cdeC*-CD0926	The reverse primer used to clone *cdeC* in the expression vector pET22b.
FP-cdeMpM11	TCTTTATTTTCAGGGCGCCATGGATATGGAAAATAAAAAATATGCAAATGGTGGTTATTCAGA	+3 to +38	*cdeM*-CD1478	The forward primer used to clone *cdeM* in the expression vector pETM11.
Rp-cdeMpM11	TGGTGGTGGTGGTGCTCGAGTTTTCTACAGCAGTTACAATTACATTTATGGCATTTATGG	-453 to -492	*cdeM*- CD1478	The forward primer used to clone *cdeM* in the expression vector pETM11.
FP-cdeApM11	TCTTTATTTTCAGGGCGCCATGGTGAAAAATAATAATTTAAATTGTGCTGCTACTAATTGTGCTTATAATACT	+3 to +51	*cdeA*-CD2262	The forward primer used to clone *cdeA* in the expression vector pETM11.
RP-cdeApM11	TGGTGGTGGTGGTGCTCGAGTTTCATTTCAAAAGTTTCACAACTTGCATTTCTTTCATTTATATGAAC	-259 to -306	*cdeA*-CD2262	The reverse primer used to clone *cdeA* in the expression vector pETM11.
FP-M1-D100 CdeC-N*de*I	GACCATATGCAAGATTATAAAAAAAAATAAAAGAAGAATGATGAATCAGC	+3 to +45	*cdeC*-CD0926	The forward primer used to clone truncated sequences of *cdeC* in the expression vector pET22b, corresponding to coding nucleotides for M1 to D100 of the complete sequence of *cdeC.*
RP-M1-D100 CdeC-X*ho*I	GACCTCGAGATCCATTTCACATGGTTCACAATCACATTTAC	-267 to -297	*cdeC*-CD0926	The reverse primer used to clone the truncated sequence of *cdeC* in the expression vector pET22b, corresponding to coding nucleotides for M1 to D100 of the complete sequence of *cdeC*
FP-M1-N214 CdeC-N*de*I	GACCATATGCAAGATTATAAAAAAAAATAAAAGAAGAATGATGAATCAGC	+3 to +45	*cdeC*-CD0926	The forward primer used to clone truncated sequences of *cdeC* in the expression vector pET22b, corresponding to coding nucleotides for M1 to N214 of the complete sequence of *cdeC.*

RP-M1-N214 CdeC-*Xho*I	GACCTCGAGGTTTCTTCC TACTATATCTCCTAATGG G	- 610 to -639	*cdeC*-CD0926	The reverse primer used to clone truncated sequences of *cdeC* in the expression vector pET22b, corresponding to coding nucleotides for M1 to N214 of the complete sequence of *cdeC*.
FP-P206-R405 CdeC-N*de*I	GACCATATGCCATTAGGA GATATAGTAGGAAGAAA CTG	+613 to +639	*cdeC*-CD0926	The forward primer used to clone truncated sequences of *cdeC* in the expression vector pET22b, corresponding to coding nucleotides for P206 to R405 of the complete sequence of *cdeC*.
RP-P206-R405 CdeC-X*ho*I	GACCTCGAGTCTGTGGCA ACTTGGCTTTCCACTTC	-1189 to -1215	*cdeC*-CD0926	The reverse primer used to clone truncated sequences of *cdeC* in the expression vector pET22b, corresponding to coding nucleotides for P206 to R405 of the complete sequence of *cdeC*.

[a] Restriction site is marked by an underline.

[B] the nucleotide position number begins from the first codon and refers to the relevant position within the respective gene sequence.

[C] Database: Accession No. FN545816.1
